# Towards Personalized Treatment and Molecular Research on Gastrointestinal Tumors

**DOI:** 10.3390/ijms241814283

**Published:** 2023-09-19

**Authors:** Alessandro Passardi, Emanuela Scarpi, Paola Ulivi

**Affiliations:** 1 Department of Medical Oncology, IRCCS Istituto Romagnolo per lo Studio dei Tumori (IRST) “Dino Amadori”, Via P. Maroncelli 40, 47014 Meldola, Italy; 2 Unit of Biostatistics and Clinical Trials, IRCCS Istituto Romagnolo per lo Studio dei Tumori (IRST) “Dino Amadori”, Via P. Maroncelli 40, 47014 Meldola, Italy; emanuela.scarpi@irst.emr.it; 3 Biosciences Laboratory, IRCCS Istituto Romagnolo per lo Studio dei Tumori (IRST) “Dino Amadori”, Via P. Maroncelli 40, 47014 Meldola, Italy; paola.ulivi@irst.emr.it

Gastrointestinal cancers (GC) account for 26% of all cancer incidences and 35% of all cancer-related deaths [1]. Despite the decrease in the incidence of some GC subtypes, this group of tumors still constitutes an important challenge for public health. Recent years have seen remarkable advances in the treatment of GC. In particular, translational research has led to significant benefits in the diagnosis and management of such tumors, and precision medicine is fast becoming the aim of scientific research. Individualized treatment for GC in both adjuvant and metastatic settings is increasingly emphasized. In particular, the introduction of molecular-targeted agents as well as immunotherapy has significantly improved patient outcome; however, predictive markers of efficacy are still lacking. Due to these new therapeutic options, physicians are confronted with new challenges, such as monitoring progression and stratifying patients for appropriate treatments.

Colorectal cancer (CRC) is the fourth leading cause of cancer mortality worldwide. Both environmental and genetic risk factors are recognized. Among genetic diseases associated with the ereditary form of CRC, Lynch syndrome and Familial Adenomatous Polyposis (FAP) are the best-known. Alongside them, the role of other syndromes is widely recognized, such as, for example Li–Fraumeni, MUTYH-associated polyposis, Peutz–Jeghers, Cowden, and Juvenile Polyposis syndrome. Moreover, Next-Generation Sequencing (NGS) techniques have led to the identification of new genes involved in the predisposition to CRC, as reviewed by F. Rebuzzi and collaborators. The authors concluded by suggesting a choice of wide NGS panels, including the genes involved in the main cancer syndromes and, possibly, the new emerging genes [2].

The study by Ismail H.T.H and collaborators explored the role of G protein-coupled receptor 55 (GPR55) as a prognostic marker in CRC patients treated with curative surgery. GPR55 acts as a regulator of innate immunity and tumor immunosurveillance, through the activation of T cells and NK cells, as shown in mice models. A new highly specific quantitative reverse transcriptase–polymerase chain reaction (qRT-PCR) assay was used to analyze the mRNA expression levels of GPR55 in 382 regional lymph nodes removed during surgical treatment from 345 patients with CRC. Unexpectedly, the authors found that low levels of GPR55 mRNA in lymph nodes were associated with poor prognosis. The GPR55 expression level was then combined with CEA mRNA levels to form the GPR55/CEA ratio, in order to improve its prognostic value. In the high-GPR55/CEA group of patients, an increased survival of 14 and 33 months compared with the low expression group when followed for 5 and 12 years (*p* = 0.0003 and *p* = 0.003), respectively. The authors concluded that that GPR55 may serve as a marker for positive prognosis, either alone or combined with CEA, in CRC [3].

The same research group conducted an interesting trial to investigate the prognostic role of mRNA expression levels of cancer stem cells markers (epithelial cell adhesion molecule, EpCAM, and leucine-rich repeat-containing G protein-coupled receptor, LGR5 and LGR4) in surgically treated CRC patients. Expression levels were determined both in primary tumors and regional lymph nodes of CRC patients. Patients with high levels of EpCAM, LGR5 or LGR4 in lymph nodes had a decreased mean survival time of 32 months for EpCAM and 42 months for both LGR5 and LGR4, at a 12-year follow-up (*p* = 0.022, *p* = 0.005 and *p* = 0.011, respectively). The prognostic value of LGR5 mRNA levels was significantly increased when combined with the measurement of CEA, CXCL17 and CXCL16 mRNA levels. The study highlights the efficacy of mRNA evaluation in regional lymph nodes to select patients with bad prognosis. Moreover, it shows the higher accuracy of qRT-PCR with respect to immunohistochemistry, and identifies the lymph nodes as a more reliable site for the evaluation than the primary tumor [4].

Pancreatic neuroendocrine tumors (pNETs) are rare tumors, representing 5% of all pancreatic tumors. At the present therapeutic and diagnostic options, as well as tools for patient stratification are very limited. Therefore, it is an unmet need to find new markers and therapeutic strategies to improve patient outcome quality of life. In the review by Havasi and collaborators the state of the art and future perspectives in the management of pNETs are analyzed, including genetic and epigenetic approaches. In particular, authors focused on miRNAs as potential prognostic, predictive, or diagnostic biomarkers and discussed their function as future therapeutic targets [5].
